# Advances in mRNA LNP-Based Cancer Vaccines: Mechanisms, Formulation Aspects, Challenges, and Future Directions

**DOI:** 10.3390/jpm14111092

**Published:** 2024-11-04

**Authors:** Eslam Ramadan, Ali Ahmed, Youssef Wahib Naguib

**Affiliations:** 1 Institute of Pharmaceutical Technology and Regulatory Affairs, University of Szeged, H-6720 Szeged, Hungary; eslam.ramdan@mu.edu.eg; 2 Department of Pharmaceutics, Faculty of Pharmacy, Minia University, Minia 61519, Egypt; 3 Department of Clinical Pharmacy, Faculty of Pharmacy, Minia University, Minia 61519, Egypt; ali.esmael@mu.edu.eg; 4 Department of Pharmaceutical Sciences and Experimental Therapeutics, College of Pharmacy, University of Iowa, Iowa City, IA 52242, USA

**Keywords:** mRNA vaccines, cancer immunotherapy, tumor, LNPs, mRNA modifications, nucleoside-modified mRNA, innate immunity, ionizable lipids

## Abstract

After the COVID-19 pandemic, mRNA-based vaccines have emerged as a revolutionary technology in immunization and vaccination. These vaccines have shown remarkable efficacy against the virus and opened up avenues for their possible application in other diseases. This has renewed interest and investment in mRNA vaccine research and development, attracting the scientific community to explore all its other applications beyond infectious diseases. Recently, researchers have focused on the possibility of adapting this vaccination approach to cancer immunotherapy. While there is a huge potential, challenges still remain in the design and optimization of the synthetic mRNA molecules and the lipid nanoparticle delivery system required to ensure the adequate elicitation of the immune response and the successful eradication of tumors. This review points out the basic mechanisms of mRNA-LNP vaccines in cancer immunotherapy and recent approaches in mRNA vaccine design. This review displays the current mRNA modifications and lipid nanoparticle components and how these factors affect vaccine efficacy. Furthermore, this review discusses the future directions and clinical applications of mRNA-LNP vaccines in cancer treatment.

## 1. Introduction

Despite decades of extensive research, cancer remains one of the leading causes of death worldwide, accounting for approximately 10 million deaths in 2022 [1]. Patient outcomes following the use of conventional cancer treatment approaches, such as chemotherapy, radiation therapy, and surgery, remain unsatisfactory, especially for advanced and metastatic tumors. Over the past years, cancer immunotherapy has attracted great attention as a new field exploiting the potential of the immune system for eradicating different kinds of tumors. Cancer immunotherapies aim to elicit host immune responses to recognize and eliminate tumor cells or counteract the suppressive tumor microenvironment (TME) and thereby lead to tumor regression and, ultimately, improved patient outcomes [2]. These goals can be achieved by different immunotherapy techniques, including adoptive cell therapy, immune checkpoint inhibitors, chimeric antigen receptor (CAR)–T cell therapy, and cancer vaccines [3,4,5,6]. Cancer vaccines are a promising immunotherapy approach that can be useful both prophylactically and therapeutically. The vaccination process involves the introduction of tumor-specific antigens (TSAs) or tumor-associated antigens (TAAs) to the patient’s immune system to induce tumor-specific cellular and/or humoral immune responses against the tumor cells that overexpress these antigens, leading to the destruction of malignant cells. The idea of therapeutic cancer vaccines dates back to 1891, when Dr. William Coley tried to treat a cancer patient by an intratumor injection of inactivated *Serratia marcescens* and *Streptococcus pyogenes* (Coley’s toxin) in an attempt to stimulate the patient’s immune system to fight the tumor. Despite early skepticism, his theory has been confirmed by current immunology [7]. The diligent work in cancer vaccine research led to the FDA approval of the first therapeutic cancer vaccine (TheraCys^®^; Sanofi, Paris, France) in 1990 for the treatment of early-stage bladder cancer [8]. PROVENGE^®^ (Dendreon Pharmaceuticals LLC, CA, USA) is another therapeutic cancer vaccine which received FDA approval in 2010 for the treatment of metastatic castration-resistant prostate cancer (mCRPC) [9]. Furthermore, IMLYGIC^®^ (Amgen Inc., CA, USA), a viral-based vaccine, was also approved by the FDA in 2015 for the treatment of advanced melanoma [10]. In addition to these successful achievements in cancer vaccination, there are multiple vaccine candidates currently being evaluated in clinical trials, including a number of mRNA-based cancer vaccines [11].

Recently, mRNA-based vaccines attracted attention as a promising and effective vaccination strategy since the approval of mRNA COVID-19 vaccines (Pfizer/BioNTech BNT162b2 and Moderna mRNA-1273) in 2020 [12]. The rapid development and approval of mRNA vaccines made it possible to carry out large vaccination campaigns during the COVID-19 pandemic, saving millions of lives around the world. This successful application of mRNA LNP-based vaccines encouraged researchers to investigate the potential of applying this effective vaccine platform in cancer immunotherapy. Typically, mRNA vaccines consist of mRNA molecules encoding a specific target antigen and a delivery system capable of transfecting the host cells. Once mRNA is taken up by the cells, it uses the cellular machinery to produce the encoded vaccine antigens intracellularly which in turn stimulate robust antigen-specific immune responses [13]. This vaccination approach outperforms traditional vaccines in terms of safety, efficacy, and rapid cost-effective manufacturing on a large scale [14].

Although the mRNA sequence is the key element in mRNA-based vaccines, the delivery system is another crucial component that must be designed carefully to ensure a high transfection efficiency and mRNA protection in the harsh internal environment. Multiple mRNA delivery systems have been extensively studied in preclinical and clinical studies including liposomes [15,16], lipoplexes [17,18], polyplexes [19,20], virus-like particles (VLPs) [21], and dendritic cells (DCs) [22,23]. However, lipid nanoparticles (LNPs) are the only delivery system that have been proved to be clinically effective and have received FDA approval for human use [24,25]. In this review, we discuss the basics of mRNA LNP-based vaccine technology including their mechanisms, design and preparation, and the possible challenges that might hinder their development and approval for cancer treatment. This review also discusses the current progress in preclinical and clinical studies in mRNA LNP-based cancer vaccines and the future directions in this particular field.

## 2. Mechanisms Behind mRNA LNP-Based Vaccine Action

**Figure 1:**
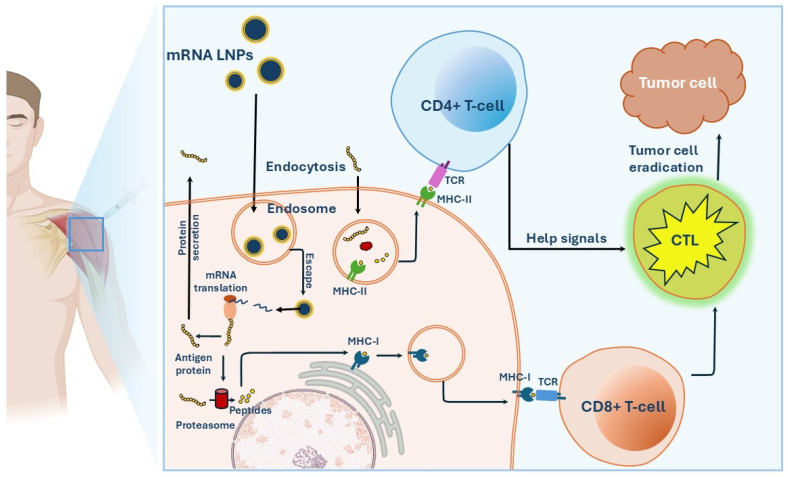
Mechanism of mRNA LNP-based cancer vaccines.

Briefly, mRNA-based cancer vaccines introduce synthetic mRNA molecules encoding the target TSAs or TAAs to the host cells. Once inside the host cells, these mRNA molecules are translated into the corresponding antigens which in turn activate antigen-specific cellular and humoral immune responses leading to the destruction of tumor cells (Figure 1) [26]. The detailed mechanism of each step will be discussed in the following sections.

### 2.1. mRNA-LNPs’ Delivery and Cellular Uptake

Since mRNA molecules are unstable, have a low membrane permeability, and are susceptible to degradation by extracellular nucleases, an efficient delivery system is required to provide mRNA protection and mediate intracellular delivery to the target cells [27]. Therefore, various formulations have been developed to serve this purpose. Amongst the most successful formulations, LNPs were the first and only RNA delivery system to receive FDA approval for human use. mRNA LNP-based vaccines are internalized mainly at the injection site by non-immune cells (muscle cells, keratinocytes, and fibroblasts) [26,28]. This internalization involves a series of well-orchestrated steps. Initially, the mRNA-loaded LNPs are administered and subsequently they approach the cell membrane to initiate LNP-cell membrane interaction. This interaction is the first step in the cellular uptake of mRNA-LNPs and is facilitated by the lipid components of the nanoparticles, which promote cellular uptake. It has been established that LNPs undergo internalization mainly by clathrin-mediated endocytosis (CME) and macropinocytosis [29,30]. Gilleron, J. et al. reported that the inhibition of key CME regulators, the clathrin-heavy chain (CLTC), and the low-density lipoprotein receptor (LDLR), led to a dramatic reduction in the extent of LNP uptake (50–60%). Likewise, the pharmacological inhibition of macropinocytosis by treating cells with ethylisopropylamiloride (EIPA) resulted in about a 70% reduction in LNP uptake. In contrast, the silencing of Caveolin 1 (CAV1), a regulator of caveolin-mediated endocytosis, had no effect on the LNP internalization. These findings suggested that the CME and macropinocytosis, but not the caveolin-mediated endocytosis, were responsible for the LNP uptake. Moreover, they highlighted that the macropinocytosis was the major mechanism of the LNP internalization; however, it was only activated after the LNP uptake by the CME, indicating that LNPs are first internalized by the means of CME followed by the activation of macropinocytosis which increases the speed and extent of LNP uptake [29].

Upon endocytosis, LNPs are internalized into different vesicular structures with a pH gradient starting from early endosomes (pH~6.3) which progress to late endosomes (pH~5.5) and finally fuse with lysosomes (pH < 5) [30,31]. To ensure the successful delivery of mRNA to the cytosol, the LNPs must escape the endosomes before fusion with the lysosomes, in which mRNA can be degraded. This process is known as endosomal escape and is considered the most critical step in the intracellular delivery of mRNA LNPs. One of the most agreed upon theories of endosomal escape emphasizes the role of ionizable lipid and/or fusogenic phospholipid components in LNPs. Once in the acidic pH of endosomes, ionizable lipids become positively charged and interact with the negatively charged lipids of the endosomal membrane. This interaction leads to the membrane fusion of the LNPs with the endosomal membrane followed by the formation of a non-bilayer structure (hexagonal H_II_), leading to the destabilization and eventually damage of the endosomal membrane and the release of mRNA to the cytosol [32,33]. Maugeri, M. et al. hypothesized that, following the endosomal membrane fusion of LNPs, mRNA molecules form a water-insoluble complex salt with the ionizable lipids (1:1) at the endosomal pH. Since a complex salt of this kind may be lipid-soluble, it may be carried across the endosomal membrane during the LNP fusion process. The complex will begin to dissociate once it reaches the cytoplasmic side of the endosomal membrane, where the pH is neutral (~7.4), producing mRNA that is available intracellularly for protein synthesis [34].

Another proposed mechanism of endosomal escape is known as the “proton sponge effect”. This hypothesis is based on the buffering capacity of the ionizable lipids used in LNPs. It postulates that, upon the entry of the LNPs and while residing in the endosomes, proton pumps on the endosomal membrane (V-ATPase) are activated to pump protons inside the endosomes in order to lower the endosomal pH. This pH lowering normally happens during endosomal maturation to produce late endosomes. However, in the presence of LNPs, ionizable lipids bind to these protons, limiting the acidification process and leading to the entry of more protons to overcome the buffering capacity of these lipids and lower the pH. In order to maintain the charge balance, the entry of more protons is accompanied by the entry of chloride ions. This increases the ionic concentration inside the endosomes and promotes the influx of water to maintain osmolarity. Finally, the entry of water leads to endosomal swelling and rupture due to the high osmotic pressure and, eventually, the release of mRNA-LNPs to the cytosol [35,36]. It is worth mentioning that our current knowledge on endolysosomal trafficking is mainly based on in vitro experimental models. Further research is needed to understand whether the in vitro observations well-represent in vivo situations. A better understanding of the mechanism can help design and optimize more efficient mRNA delivery systems with an improved transfection efficiency and a lower risk of adverse effects.

### 2.2. Antigen Expression and Presentation

Once in the cytoplasm, mRNA is translated by the ribosomal machinery, enabling the production of the encoded antigen protein. The synthesized antigen protein is either secreted into the extracellular environment or degraded intracellularly by the action of proteasomes and trafficked to the rough endoplasmic reticulum (rough ER). The secreted antigens can undergo cellular uptake by the same cell or by other adjacent cells and localize in the endosomes where they can be processed and degraded into antigen peptides. These antigen peptides are then associated with the major histocompatibility complex (MHC), resulting in the formation of MHC–peptide complexes. These complexes are then transported to the cell surface, where they can be presented to T lymphocytes [37].

There are two types of MHC molecules involved in antigen presentation to T cells: MHC class I and class II. MHC I molecules are responsible for presenting epitopes derived from intracellular antigens such as viral proteins and intracellular bacteria. Therefore, they are localized in the cytoplasm of most cells where they can bind peptides before being expressed on the cell surface [38]. Once bound by MHC I molecules, the peptide epitopes can be expressed on the surface of APCs in the form of the peptide–MHC I complex which can be recognized by T cell receptors (TCRs) on CD8+ T cells. Epitope presentation to CD8+ T cells by MHC I molecules is responsible for the activation of cytotoxic T lymphocytes (CTLs) that mediate the elimination of tumor cells within the context of cellular immunity [39]. On the other hand, MHC II molecules are responsible for presenting epitopes derived from extracellular antigens such as bacterial and viral proteins as well as non-self-cellular proteins. Therefore, they are present in the endosomes and lysosomes of APCs where foreign antigens are processed into peptides before their insertion into the binding groove of MHC II molecules [40]. These peptide epitopes can then be expressed on the surface of APCs in the form of the peptide–MHC II complex which can be recognized by TCRs on CD4+ T cells.

### 2.3. Immune System Activation

In general, mRNA LNP-based vaccines have been designed to induce antigen-specific adaptive immune responses, responsible for the eradication of tumor cells or pathogens. However, cumulative studies suggest that mRNA-LNPs can also induce innate immunity.

#### 2.3.1. Innate Immune Response

Innate immunity is the non-specific immune response elicited to detect pathogens and stimulate inflammatory reactions. It is important for the initiation of adaptive immunity by providing signals for T cell activation and DCs maturation. In mRNA vaccines, innate immunity is induced directly by mRNA molecules after being recognized by different pattern recognition receptors (PRRs), which initiate complex intracellular signaling cascades. PRRs are located in endosomes or the cytosol, allowing pathogen-associated molecular patterns (PAMPs) to be detected at two distinct cellular levels. Toll-like receptors (TLRs) identify exogenous mRNA within endosomes: TLR3 recognizes double-stranded RNA (dsRNA) with lengths of 40–45 base pairs while TLR7 and TLR8 detect single-stranded mRNA (ssRNA) rich in GU regions [13,41]. Cytosolic PRRs include melanoma differentiation-associated protein 5 (MDA-5), retinoic acid-inducible gene I (RIG-I), nucleotide-binding oligomerization domain 2 (NOD2), and RNA-dependent protein kinase (PKR). MDA-5 recognizes dsRNA longer than 2 kb, and RIG-I detects 5′-triphosphate short dsRNA, while NOD2 can recognize ssRNA, leading to the activation of interferon-regulatory factor 3 (IRF3) and the production of type I interferons [42]. These PRRs typically activate the type I interferon pathway that is critical for the CD8+ T cell response. Type I interferon activation also leads to the secretion of interferons α and β, which upregulate PKR and oligoadenylate synthetase (OAS) [43,44,45,46]. PKR, a serine–threonine kinase and dsRNA sensor, inhibits mRNA translation by phosphorylating eukaryotic initiation factor 2 (eIF2). OAS, also a dsRNA sensor, activates RNase L to degrade mRNA [37]. Consequently, if in vitro-transcribed (IVT) mRNA is identified as exogenous, both the translation of the target protein and its therapeutic efficacy are significantly reduced.

In addition to mRNA molecules, ionizable LNPs can induce a robust innate immune response. Recent studies have indicated that LNPs not only serve as delivery vehicles but also possess intrinsic adjuvant properties that enhance immune responses. For example, Alameh MG. et al. reported that the intramuscular (IM) and intradermal (ID) administration of influenza virus hemagglutinin recombinant protein (rHA) with empty ionizable LNP formulation induced robust humoral responses higher in magnitude than those induced by rHA protein adjuvanted with the AddaVax adjuvant. These results suggested that LNP formulation possesses strong adjuvant activity. Remarkably, the adjuvant effect of empty LNPs was eliminated by removal of the ionizable lipid component from the formulation and enhanced by increasing their molar ratio, indicating the critical role of ionizable lipids in activating innate immunity [47]. At present, the detailed signaling pathway involved in the activation of innate immunity by ionizable LNPs is still unclear. However, studies have shown that empty ionizable LNPs induce the production of cytokines including interleukin-6 (IL-6), interleukin-1β (IL-1β), granulocyte–macrophage colony-stimulating factor (GM-CSF), and multiple chemokines [48,49]. Ndeupen et al. also reported the upregulation of the *Nlrp3* gene, associated with inflammasome activation, and the downregulation of inflammasome inhibitory *Nlrp10* after the ID injection of LNPs, signifying the contribution of NLRP3 in LNPs’ recognition which leads to secretion of IL-1β, a potent proinflammatory cytokine [49]. Other studies suggest that cationic or ionizable lipid components in LNPs can be recognized as danger-associated molecular patterns (DAMPs) by PRRs such as TLRs. Cationic lipids can activate extracellular membrane-associated TLR2 and TLR4 which mediate production of high levels of proinflammatory cytokines including IL-6 [50,51,52,53]. Moreover, Miao, L. et al. designed new heterocyclic ionizable lipids, containing cyclic amine headgroup, to improve the antitumor efficacy of mRNA-LNP vaccines. Interestingly, LNPs containing cyclic ionizable lipid (A18) demonstrated an adjuvant effect mediated by the stimulator of the interferon genes (STING) pathway instead of TLR signaling. mRNA-LNP formulations containing this cyclic lipid showed potent antitumor activity in melanoma and human papillomavirus E7 tumor models, even better than MC3-based LNPs [54].

Besides the direct sensing of mRNA and LNP components, mRNA-LNPs have been reported to induce inflammation by causing damage to the endosomal membrane. Omo-Lamai S. et al. demonstrated that the large, irreparable endosomal membrane holes resulting from the endosomal escape process can be detected by a set of cytosolic proteins called galectins. These proteins bind to sugars on the exposed inner endosomal membrane and initiate downstream signaling pathways leading to the innate immune response. The administration of galectin inhibitors eliminates LNP-induced inflammation. Moreover, the use of rapidly biodegradable ionizable lipids in LNPs is associated with creating smaller endosomal holes that can be repaired by the endosomal sorting complex required for the transport (ESCRT) pathway, with a subsequent reduction in LNP-induced inflammation [55].

Additionally, the PEG component in LNPs can induce the production of anti-PEG antibodies, raising concerns regarding both safety and efficacy. The presence of PEG coating can trigger the production of these antibodies after the first dose, leading to the binding and rapid elimination of subsequent doses of PEGylated nanocarriers, a phenomenon known as the accelerated blood clearance (ABC) effect. This phenomenon has been reported in PEG-conjugated substances and PEGylated nanocarriers including PEGylated liposomes, PEG-conjugated proteins, PEGylated nanoparticles, and PEGylated micelles [56,57]. However, it has been recently reported that the immunogenicity of ionizable LNPs stems from the binding of the amine headgroups of ionizable lipids to TLR 4 and CD1d. The inflammatory signals triggered by these receptors can inhibit the formation of anti-PEG IgM antibodies, preventing the ABC and loss of the efficacy of the LNP-mediated delivery of mRNA [58]. Therefore, the presence of ionizable lipids in LNPs can be beneficial for two reasons: first, they can serve as an adjuvant and eliminate the need for conventional vaccine adjuvants which have their own potential adverse effects; and second, they may inhibit the production of anti-PEG antibodies, preventing the ABC phenomenon and consequently preserving the vaccine efficacy. Understanding the mechanisms of innate immune activation by mRNA-LNPs can lead to the design of self-adjuvanted LNPs with limited systemic inflammation and better therapeutic outcomes.

#### 2.3.2. Adaptive Immune Responses

mRNA-LNP cancer vaccines have been designed to induce a strong immune response against tumor cells through the introduction of mRNA encoding TAAs or TSAs to APCs. These antigens, after translation, processing, and presentation on the APC surface by MHC molecules, are recognized by CD8+ and CD4+ T cells. More specifically, the CD8+ T cells recognize their antigens complexed with MHC class I molecules using their TCRs. After the antigens bind to the TCR, T cells proliferate and differentiate into cytotoxic T lymphocytes capable of targeting and killing tumor cells. T cell activation is also augmented by costimulatory signals from APCs and a third signal provided by certain cytokines [59]. The costimulatory signal is known to be unspecific to the antigen and can be produced by molecular interactions between molecules on the surface of APCs and T cells including CD28 and CD40 pathways [60]. Moreover, inflammatory cytokines, mainly IL-12 and Type I IFN (IFNα/β), are reported to be essential for T cell activation and are called the third signal [61,62]. Activated CTLs in the context of cellular immunity can target and destroy cancerous cells expressing the target antigens. The effector cells mediate their cytotoxic action through the release of perforin and granzymes, which induce apoptosis in target cells [63]. In addition, upon successful activation, active T cells can form memory T cells that can survive for years in the body. These memory cells are crucial in mounting fast and vigorous responses whenever the same antigens are recognized again, hence reducing the risk of tumor recurrence by providing long-lasting protection.

On the other hand, antigens complexed with MHC class II molecules are presented to CD4+ T cells, which are known to mediate the activation of B cells and humoral immune response. However, the role of CD4+ T cells in tumor immunity extends beyond B cell activation. CD4+ T cells were reported to play a key role in optimal CD8+ T cell activation and providing help signals necessary to optimize CTL response [64]. Studies have shown that antitumor vaccines containing only short MHC class I-restricted peptides led to CD8+ T cell tolerance and failed to induce CTL antitumor immunity unless provided with help from the CD40 agonist antibody [65]. Another study demonstrated that a DCs-based vaccine loaded with MHC class I-restricted peptide could only induce CTL priming when DCs were stimulated in vitro with antigen-specific CD4+ T cells or CD40 agonists [66]. These findings confirm that CD4+ T cells are essential for the proper CTL response and, hence, vaccination with both MHC I and II epitopes, which activate both CD8+ and CD4+ T cells, could be more effective in inducing antitumor CTL immune responses.

## 3. Design and Formulation of mRNA LNP-Based Cancer Vaccines

### 3.1. Antigen Selection

In the development of mRNA-based cancer vaccines, proper antigen selection is the key for the efficient targeting of tumor cells. The two major kinds of antigens that are being considered for antitumor vaccines are TAAs and TSAs. These antigens can be the result of abnormal gene expression of normal genes, mutated cellular genes, or genes encoding viral proteins. All of them are expressed by tumor cells and can be used as targets for the induction of the antitumor immune response.

#### 3.1.1. Tumor-Associated Antigens (TAAs)

TAAs are proteins highly expressed in tumor cells compared to normal cells. However, they are expressed at lower levels in healthy tissues. That way, this characteristic implies that despite the TAAs can be effective antigens for cancer vaccines, they are also a potential risk factor for autoimmune reactions resulting from their expression on normal cells [67]. The identification of TAAs involves the analysis of tumor biopsies for the overexpression of certain antigens. For example, mRNA vaccines can be designed to encode particular TAAs that are relevant to a patient’s cancer type to stimulate an immune response against such antigens. Examples of TAAs include human epidermal growth factor receptor 2 (HER2), expressed in various epithelial tumors; mucin 1 (MUC-1), overexpressed by more than 70% of cancers; the carcinoembryonic antigen (CEA), overexpressed in a wide range of carcinomas, including pancreatic, colorectal, gastric, non-small-cell lung, and breast carcinomas; human telomerase reverse transcriptase (hTERT); the Tn, TF, and sialyl-Tn (STn) antigens; Wilms’ tumor gene (WT1), expressed by different cancer cells; the KRAS mutations G12D, G13D, G12C, and G12V; and melanoma-associated antigens (MAAs) [68,69]. Wang, X. et al., reported that mRNA vaccines encoding the KRAS G12V antigen in combination with a PD-1 inhibitor could induce tumor shrinkage in two end-stage cancer patients with advanced pancreatic head cancer and advanced non-small-cell lung cancer (NSCLC) [70].

#### 3.1.2. Tumor-Specific Antigens (TSAs)

**Figure 2:**
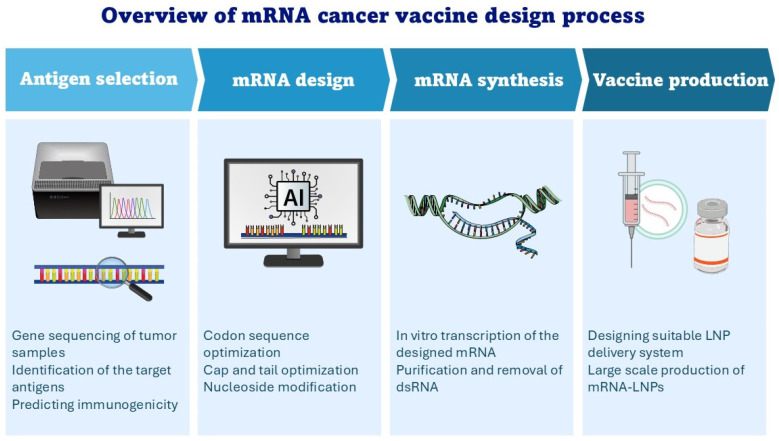
The process of mRNA cancer vaccine design.

TSAs are solely exclusive to cancer cells and cannot be detected in normal tissues. These antigens, also called neoantigens, result from mutations in tumor cells, leading to the generation of abnormal proteins that can be recognized by the immune system. Neoantigens can result from a variety of DNA mutations including point mutations, gene fusions, insertions, deletions, and frameshift mutations. However, point mutations are the most frequent in cancer cells, and can be used as neoantigen candidates [71]. TSAs offer a more precise target for the development of cancer immunotherapy because they are unlikely to lead to autoimmune-related side effects. In these regards, mRNA vaccines can be designed to encode neoantigens specific for the mutations of a patient’s tumor [72]. The identification of tumor-specific neoantigens is a lengthy process that starts with the identification of all types of the somatic mutations of tumor samples by comparing the DNA sequences of tumor cells to those of normal cells. Next, these mutations are refined using bioinformatics and machine-learning approaches to select the neoantigen peptides which have the ability to bind MHC (class I and II) molecules in high affinity and stimulate epitope-specific T cells (CD4+ and CD8+) [71]. Finally, mRNA sequences encoding these peptides are designed and synthesized in vitro to be loaded into LNP delivery systems, making up the mRNA LNP vaccine candidate for preclinical and clinical evaluations (Figure 2).

#### 3.1.3. Machine-Learning Algorithms for Epitope Prediction

Recent advances in machine learning (ML) and artificial intelligence (AI) have revolutionized various biological fields, including genomics, proteomics, and drug design, by enabling the analysis of complex data and improving the prediction of biological outcomes [73,74,75]. In the context of mRNA LNP-based cancer vaccines, ML approaches are currently being applied in predicting immunogenic epitopes which are likely to induce a robust T cell response. Classically, epitope identification has been a function of labor-intensive experimental methods. Currently, ML models enable high-speed epitope prediction by using large genomic and proteomic datasets. Many such models leverage deep learning algorithms to predict which tumor-derived antigens have the highest chance of binding to MHC molecules and, consequently, eliciting a T cell-mediated immune response [76]. Using these ML-powered techniques, Lingeng and colleagues designed mRNA vaccine candidates targeting the CA-125 neoantigen in ovarian and breast cancers. They used the NetMHCpan v4.0 model to predict MHC-I-binding peptides in somatic mutations of CA-125 breast carcinoma or ovarian cancer datasets. For the CTL epitope prediction, they used the NetCTL v1.2 model which integrates proteasomal cleavage, MHC-I affinity, and transport efficiency to HLA molecules. Using these algorithms, they identified six mutated epitope peptides in the breast cancer and three in the ovarian cancer, which met all the selection criteria for the CTL epitope vaccine construction. Additionally, the authors developed an in silico pipeline to design a self-adjuvant mRNA vaccine encoding these identified epitopes together with CD40L and MHC-I targeting domains to enhance the cross-presentation of neoepitopes by dendritic cells. Finally, the study employed the ImmSim algorithm for predicting immune responses following vaccination and found that the designed vaccine may elicit significant CD8+ T cell and IFN-γ responses, which are essential for effective anti-tumor immunity [77]. The findings of this study demonstrate that in silico vaccine design approaches can enhance both speed and precision in epitope prediction to facilitate the identification of neoantigens. This application of ML also improves the efficiency of vaccine development by reducing experimental screening to a minimum, thus opening up avenues for more effective and targeted immunotherapies.

### 3.2. Structure and Modifications of mRNA

The mRNA sequence is the key element of mRNA-based vaccines, which is responsible for the intracellular production of the desired antigen for the subsequent induction of an antigen-specific immune response [78]. Eukaryotic mRNA is a single-stranded molecule composed of ribonucleotides, which include a ribose sugar, a phosphate group, and one of four nitrogenous bases: adenine (A), guanine (G), cytosine (C), and uracil (U) [79]. The eukaryotic mRNA is capped at its 5′-end by a 7-methylguanylate cap (m7G). This cap protects the mRNA from degradation, assists the binding of ribosomes during translation, and facilitates the export of mRNA from the nucleus [80]. In addition to these, mRNA includes two other major untranslated regions: a 5′ UTR and a 3’ UTR. These regions are not meant to be translated into proteins; however, they are very important in controlling translation efficiency, mRNA stability, and intracellular localization. The 5′ UTR is upstream and the 3’ UTR is downstream to the coding sequence [78]. The mRNA coding region, also referred to as the open reading frame (ORF), is composed of codons, which are sequences of three nucleotides that encode specific amino acids. This region is flanked by the two UTRs and encodes the protein. At the 3’-end of the mRNA, a poly(A) tail, composed of adenine nucleotides, is added post-transcriptionally. This poly(A) tail stabilizes the mRNA, serves as a transcription terminator, and signals for the mRNA export from the nucleus [81].

The characteristics of unmodified mRNA such as its susceptibility to degradation by extracellular ribonucleases (RNases) and intrinsic immunogenicity limit its use in mRNA-based vaccines. Its rapid degradation can lead to a significantly reduced half-life of the mRNA once administered, limiting its ability to produce the desired immune response. Meanwhile, its intrinsic immunogenicity can lead to severe inflammatory reactions and reduce the translation efficiency, leading to a suboptimal immune response. To overcome these problems, researchers introduced modified mRNA molecules with different modifications including base modifications and cap and tail modifications, as well as codon sequence optimization (Table 1). Until recently, more than 330 natural and 94 unnatural modifications have been reported on the MODOMICS database in all types of RNA molecules [82]. Base modifications can be conducted at any of the nucleotide bases in mRNA by chemical or enzymatic reactions. For instance, uridine replacement with pseudouridine (Ψ) is one of the earliest modifications employed in mRNA vaccine research. Ψ is a natural isomer of uridine present in all organisms in a ratio of 7–9% of all uridine bases. RNA pseudouridylation is carried out by pseudouridine synthase enzymes (PUS) [78]. Karikó and co-workers reported that incorporation of Ψ in mRNA improved the translational capacity of the encoded proteins compared to the unmodified mRNA. Moreover, the delivered Ψ-modified mRNA did not induce the production of the proinflammatory cytokines IFN-α and TNF-α compared to the unmodified mRNA, indicating the nonimmunogenic nature of Ψ-modified mRNA [83]. Pseudouridine modification is thought to make mRNA undetectable by TLRs and PKR, leading to a reduced mRNA immunogenicity and improved translation efficacy. Another significant uridine modification is N1-methylpseudouridine (m1Ψ) which has been successfully utilized in COVID-19 mRNA vaccines to increase protein output and decrease TLR3 activation [84]. A study by Oliwia Andries et al. highlighted the effect of N1-methylpseudouridine (m1Ψ) modification on gene expression and the immunogenicity of mRNA. Surprisingly, m1Ψ-modified mRNA, with or without 5-methylcytidine (m5C) modification, outperformed the Ψ-modified mRNA in gene expression and led to reduced innate immunogenicity upon in vitro transfection, suggesting that m1Ψ can be a better approach for nucleoside-modified mRNA-based therapeutics [85].

**Figure 3:**
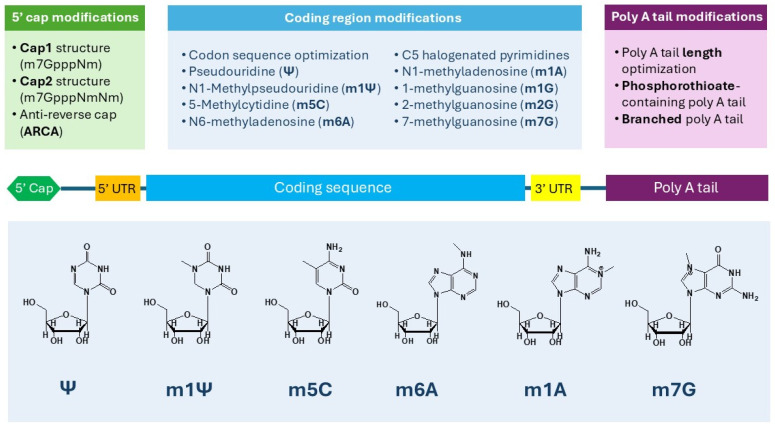
mRNA structure and nucleoside modifications.

Other modifications can be obtained by RNA methylation at different modification sites including m5C, N1-methyladenosine (m1A), N6-methyladenosine (m6A), 1-methylguanosine (m1G), 2-methylguanosine (m2G), and 7-methylguanosine (m7G) (Figure 3) [78]. Amongst them, m5C is commonly preferred as it can increase translation efficiency while lowering immunogenicity [86]. In addition, other unnatural nucleotide modifications are being investigated for their potential to further improve mRNA performance. For instance, Singh and co-workers have recently shown that C5 halogenated pyrimidines can improve protein expression. They reported that fluorocytidine- and chlorocytidine-modified mRNA had four to five times higher protein expression compared to the unmodified mRNA. Furthermore, the dual modification of mRNA with fluorocytidine and Ψ resulted in a dramatic increase in protein expression as much as tenfold [87]. All these modifications aim to address the limitations of natural unmodified mRNA for mRNA-based vaccines. Despite the use of nucleoside-modified mRNA having shown promise in improving mRNA stability and immunogenicity, further research is still needed to fully understand the impact of each modification on the performance of mRNA in mRNA-based vaccines.

**Table 1:** mRNA modifications and their potential therapeutic applications.

Modification Site	Modification	Modification Method	Function	Application	Reference
5′ Cap	Cap 1 (m7GpppNm)	Enzymatic capping	Enhances translation initiation by promoting recognition by the eukaryotic initiation factor 4E (eIF4E) and protects mRNA from degradation by exonucleases.	COVID-19 mRNA vaccines	[80]
5′ Cap	Anti-Reverse Cap Analogs (ARCAs)	Chemical synthesis	Improves the translation efficiency and prevents mRNA degradation by blocking the activity of decapping enzymes.	mRNA vaccine research	[88]
Poly(A) Tail	Branched Poly(A) Tails	Chemical synthesis	Improves the translation capacity and enhances mRNA stability by protecting the 3′ end from degradation.	mRNA therapeutics and mRNA vaccine research	[89]
Nucleoside	N1-Methylpseudouridine (m1Ψ)	Enzymatic methylation	Improves protein expression and reduces immunogenicity by mimicking the structure of eukaryotic mRNA, thereby avoiding recognition by TLRs (TLR3).	COVID-19 mRNA vaccines	[84,85,90]
Nucleoside	Pseudouridine (Ψ)	Enzymatic isomerization	Enhances translation efficiency by promoting codon–anticodon interactions and reduces immunogenicity by avoiding recognition by TLRs.	mRNA vaccine research	[83]
Nucleoside	5-Methylcytidine (m5C)	Enzymatic methylation	Enhances translation efficiency and reduces innate immune responses.	COVID-19 mRNA vaccine candidates, and mRNA vaccine research	[91,92]
Nucleoside	N6-Methyladenosine (m6A)	Enzymatic methylation	Inhibits innate immunity to RNA by mimicking the structure of eukaryotic RNA.	Potential for future mRNA vaccine development	[93]

Aside from nucleotide modifications, synthetic mRNA molecules can be modified at the cap and tail regions to improve their stability and translation efficiency. Eukaryotic mRNA is capped at the 5′ end with a 7-methylguanosine (m7G) moiety attached to the first nucleotide of mRNA through a triphosphate bridge forming a cap 0 structure (m7GpppN) [80,94]. The 5′ cap on mRNA plays a crucial role in translation initiation and mRNA stabilization by recruiting initiation factors such as eIF4G and RNA helicase eIF4A to the end of the mRNA and protecting mRNA from degradation by exonucleases. This cap structure also interacts with poly(A)-binding protein PABP1 to create a pseudo-circular structure of translating mRNA, enhancing its translation efficiency [95]. Cap modifications such as cap 1 (m7GpppNm) and cap 2 (m7GpppNmNm) structures, which have methylated nucleotides, can reduce mRNA immunogenicity compared to cap 0; therefore, the cap 1 structure has been utilized by Pfizer-BioNTech and Moderna in their mRNA-based COVID-19 vaccines [80,94]. During the in vitro synthesis of mRNAs, the cap moiety can be inserted in a reversed way in which the m7G is directly attached to the mRNA. This reverse cap can significantly reduce the translation efficiency of the synthetic mRNA. Therefore, anti-reverse cap analogs (ARCAs) are used to prevent reverse cap incorporation [96].

In addition to cap modifications, multiple poly(A) tail optimizations have also been explored to enhance mRNA stability and translation efficiency. The length of the poly(A) tail affects both mRNA stability and translation efficiency. It has been reported that a poly(A) tail of 120 nucleotides had a higher RNA stability and translational efficiency compared to a shorter one [97]. Therefore, optimizing the length of the poly(A) tail in synthetic mRNA can be a crucial factor in maximizing protein expression levels. Other chemical modifications in mRNA poly(A) tails such as the chemical modifications of 5′,3′-phosphodiester bonds have also been shown to improve mRNA stability as phosphorothioate-containing poly(A) tail could significantly reduce mRNA degradation by 3′-deadenylase compared to the unmodified mRNA with no effect on protein expression [98]. Furthermore, Hongyu Chen et al. have recently introduced a new branched poly(A) tail modification that successfully improved the translation capacity by 4.7–19.5 folds higher than the unmodified mRNA [89]. These advancements in mRNA modifications highlight the importance of tailoring the poly(A) tail to enhance protein expression. The development of new modifications continues to offer promising strategies for optimizing mRNA stability and translation efficiency.

Codon optimization is another approach that can be combined with mRNA modifications to enhance protein expression levels and control the innate immune activation. A certain amino acid can be encoded by multiple codons, and replacing the less frequently used codons with those that are more common in the target organism, can significantly improve the stability and translation efficiency of mRNA [99,100]. It has been reported that high-GC-content mRNA had a 100-fold higher translation than low GC sequences, and low uridine content or modified uridine analogs had lower immunostimulatory effects [101,102]. Thus, optimizing the mRNA codon sequence can have significant effect on the in vitro-transcribed mRNA in terms of stability, translation efficiency, and innate immune system activation. Recently, great advances in computational techniques and machine learning have led to the emergence of multiple mRNA design algorithms that can predict the most optimal codon usage. For instance, the LinearDesign algorithm, created by He Zhang and his colleagues, could find an optimal design for mRNA encoding SARS-CoV-2 spike protein (has 2.4 × 10^632^ possible mRNA sequences) in only 11 min. The optimized mRNA showed an improved stability and protein expression and led to a 128 times increase in antibody titer in mice compared to the codon optimization used in COVID-19 mRNA vaccines [103]. Moreover, a large language model (LLM) called CodonBERT has been developed and properly trained to predict mRNA sequences based on different mRNA properties [104]. These advancements in codon optimization tools demonstrate the potential for significant improvements in protein expression and vaccine efficacy through enhanced mRNA design.

Taken together, mRNA modifications and codon optimizations can greatly enhance the effectiveness of mRNA vaccines by improving stability, protein expression, and the innate immune response. Therefore, a successful mRNA cancer vaccine should carefully incorporate these advancements in mRNA design to maximize its therapeutic potential and effectiveness.

### 3.3. LNPs as an Efficient mRNA Delivery System

**Figure 4:**
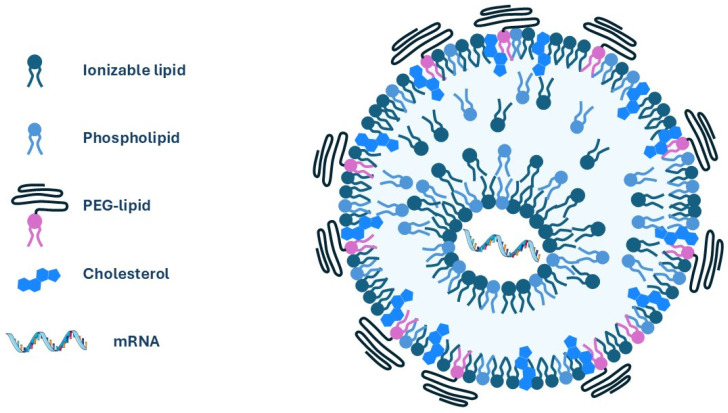
Lipid nanoparticle components.

Currently, lipid nanoparticles (LNPs) are considered the most advanced delivery system for mRNA vaccines, due to their ability to protect mRNA from degradation and efficiently deliver it into cells. LNPs have shown promising results in preclinical and clinical studies, making them a key component in the development of effective mRNA cancer vaccines [105]. Initially, LNPs had emerged as a delivery system for small interfering RNA (siRNA) therapeutics and received FDA approval for the first time in August 2018 [106]. Since then, they have proven effective for the delivery of mRNA vaccines and have played a crucial role in the rapid development of COVID-19 vaccines, as they have the potential to induce robust immune responses due to their high transfection efficiency and intrinsic adjuvant activity [107,108]. LNPs are small lipid-based nanocarriers with a size of around 100 nm in diameter, which are usually composed of a cationic ionizable lipid and other helper lipids, including phospholipids, cholesterol, and a polyethylene glycol-conjugated lipid (PEGylated lipid) (Figure 4) [109]. The type and composition of lipids in LNPs can affect different LNPs’ properties such as their physicochemical properties, biodistribution, cellular uptake, endosomal escape, and immunogenicity [109]. Therefore, the careful design and optimization of LNPs is a crucial step to unlock the full potential of mRNA-based cancer vaccines.

Cationic lipids such as 1,2-di-O-octadecenyl-3-trimethylammonium-propane (DOTMA) and 1,2-dioleoyl-3-trimethylammonium-propane (DOTAP), which have a permanent positive charge on their head groups, have been extensively studied for mRNA delivery in combination with other lipids [16,110,111,112]. The positive charge on the lipid head group mediates electrostatic interaction with the negatively charged nucleic acid molecules, securing a high mRNA encapsulation efficiency [113]. However, the use of such permanent cationic lipids for nucleic acid delivery is associated with cellular toxicity and a low therapeutic efficacy which limits their application in mRNA-based vaccines [114,115,116]. Over the past two decades, multiple ionizable lipids have been developed to overcome the charge-induced toxicity of the earlier cationic lipids and to improve the transfection efficiency of LNPs [117]. Ionizable lipids were designed to gain a positive charge only at low pH conditions, during LNPs preparation and in the endo-lysosomal compartments after cellular internalization, while being neutral at physiological pH conditions to limit the charge-related toxicity [117]. The positive charge of ionizable lipids is responsible for the electrostatic interaction with negatively charged nucleic acids during the LNPs’ preparation, as well as the endosomal escape of the loaded cargo through endosomal membrane fusion after cellular internalization, which allows the payload to be efficiently delivered to the cytosol of the target cells [109,117]. The first ionizable lipid to be reported was [3-(Dimethylamino)-2-octadec-9-enoyloxypropyl] octadec-9-enoate (DODAP) [118], followed by the development of 1,2-dilinoleyloxy-n,n-dimethyl-3-aminopropane (DLinDMA) and 2-[2,2-bis[(9Z,12Z)-octadeca-9,12-dienyl]-1,3-dioxolan-4-yl]-N,N-dimethylethanamine (DLin-KC2-DMA), also known as KC2, which greatly improved the transfection efficiency and therapeutic window [119]. The rational design of ionizable lipids for siRNA delivery resulted in the ionizable lipid 4-(dimethylamino)-butanoic acid (6Z,9Z,28Z,31Z)-heptatriaconta-6,9,28,31-tetraen-19-yl-4-(dimethylamino) butanoate (DLin-MC3-DMA), known as MC3, which has been used in the FDA approved siRNA therapeutic Onpattro^®^ [119]. Recently, multiple ionizable lipids have been developed for LNPs intended for mRNA delivery; two of them have received approval for clinical use in COVID-19 mRNA vaccines, namely, 8-[(2-hydroxyethyl)-6-[6-oxygen(11 alkyl hexyl)amino]-1-octylnonyl octylic acid ester] (SM-102), developed by Moderna, and 2-hexyl octylic acid 1,1′-[[(4-hydroxy-2,6-diisopropylphenyl)amino]adipic] acetate (ALC-0315), designed by Pfizer/BioNTech [120,121]. Other ionizable lipids are being designed and studied for various purposes including mRNA cancer vaccines. For example, heterocyclic ionizable lipids have been developed for anti-tumor mRNA vaccines. These lipids, characterized by cyclic amine head groups, a dihydroimidazole linker, and an unsaturated lipid tail, could stimulate APCs’ maturation through the STING pathway, resulting in an improved anti-tumor efficacy with minimal systemic inflammation [54].

Meanwhile, helper lipids play an important role in the formation and stabilization of LNPs by providing structural integrity and controlling their physicochemical properties. Typical helper lipids include phospholipids, cholesterol or cholesterol derivative, and PEGylated lipids. Phospholipids such as 1,2-dioctadecanoyl-sn-glycero-3-phosphocholine (DSPC) and 1,2-dioleoyl-sn-glycero-3-phosphoethanolamine (DOPE) are the most common helper lipids used in LNPs’ formulation. The selection of helper lipids can affect the in vivo performance of the LNPs system, especially the biodistribution and endosomal escape properties. It has been reported that DOPE-containing LNPs preferentially accumulate in the liver, while DSPC-containing LNPs exhibit higher accumulation in the spleen [122,123,124]. Cholesterol and PEGylated lipids play an essential role in maintaining the physical stability of particles. Cholesterol incorporation within the outer shell of the LNPs is important for controlling the membrane rigidity and compactness which in turn reduces the payload leakage from the LNPs’ core [119]. PEGylated lipids have an important role in LNPs’ stability as they prevent particles’ aggregation during preparation and storage in addition to prolonging the circulation half-life of LNPs’ preparations [24,125].

The molar ratio between the different lipid components is also a critical parameter that determines the physicochemical properties and transfection efficiency of LNPs. The clinically approved Pfizer-BioNTech COVID-19 vaccine has a lipid composition of ALC-0315 (46.3%), DSPC (9.4%), cholesterol (42.7%), and ALC-0159 (1.6%), while Moderna’s COVID-19 vaccine uses SM-102, DSPC, cholesterol, and PEG2000-DMG, in a molar ratio of 50:10:38.5:1.5 [24]. However, other lipid ratios are being studied by researchers to further optimize mRNA delivery and improve the immune response [126,127,128,129].

LNPs can be prepared by different methods such as microfluidic mixing, pipette mixing, and vortex mixing [130,131,132,133]. Microfluidic mixing is the most common preparation method, which involves the rapid mixing of the lipid and mRNA solutions within a microfluidic device, typically a microchannel mixer or a T junction. In this method, the lipid components are dissolved in ethanol and the mRNA is dissolved in a suitable aqueous buffer, and the two solutions are mixed together in the microfluidic device in a controllable flow rate and ratio under a controlled temperature. This method allows for precise control over the mixing parameters, leading to the formation of more homogeneous and reproducible LNP formulations [130,134]. Pipette and vortex mixing methods are simple methods commonly used for small- and medium-scale laboratory batch productions. They lack precise control over LNPs’ properties and can produce relatively larger particle sizes compared to microfluidic mixing. In pipette mixing, the organic and aqueous solutions are mixed manually by constantly and rapidly pipetting up and down to allow the nanoparticle formation. This method is easy, cost-effective, and suitable for the small-scale preparation of volumes below 100 µL. Vortex mixing is another simple method involving the mixing of the organic and aqueous solutions by vigorous vortexing until LNPs are formed. This method is also easy and requires only an inexpensive vortex mixer and can be used to prepare sample volumes below 200 µL [132]. Other conventional methods can also be used for LNP preparation, such as the thin film hydration and ethanol injection methods, which are widely applied in the preparation of liposomes.

LNPs play a crucial role in mRNA-based cancer vaccines by facilitating the efficient delivery of mRNA to target cells, necessitating specific design and optimization. For example, lipid components such as ionizable lipids, phospholipids, cholesterol, and PEG-lipids, along with preparation parameters like the mixing speed, mixing ratio, lipid-to-mRNA ratio, and temperature, can influence the efficacy of LNPs in delivering mRNA vaccines. Therefore, the study and optimization of these factors should be seriously considered in the design of mRNA cancer vaccines.

### 3.4. Manufacturing and Scalability

The large-scale production of mRNA LNP-based vaccines is a challenging process with many factors that must be controlled to ensure batch-to-batch consistency, product quality, and vaccine efficacy. The production process involves two distinct steps; the synthesis of high-purity mRNA through in vitro transcription and the encapsulation of mRNA in the LNP delivery system. Each step must be tightly controlled to avoid batch variability, degradation, or contamination [135].

mRNA is synthesized in a cell-free medium in a series of manufacturing steps that can be categorized into upstream and downstream processes. Upstream processes involve the enzymatic synthesis of mRNA from the DNA template using RNA polymerases. First, the DNA template is designed, synthesized, and purified. DNA templates can be designed in the form of double-stranded oligonucleotides, PCR products, or plasmid DNA. These templates must contain a suitable promoter sequence to provide a recognition site for the RNA polymerase during the in vitro transcription of mRNA. The T7 promoter sequence and T7 RNA polymerase are the most common promoter/polymerase system used in the large-scale production of mRNA [136,137]. The manufacturing of template DNA can be achieved through bacterial fermentation or enzymatic synthesis techniques such as PCR. In the bacterial fermentation technique, *Escherichia coli* (*E. coli*) strains are transformed with the designed DNA plasmids, and then bacterial culture expansion is carried out in either a large-scale bioreactor or through shake flask fermentation. DNA production by this method, besides being costly and time-consuming, may suffer an increased risk of bacterial contamination. Therefore, cell-free and time-efficient synthetic DNA approaches are being applied to overcome these challenges [137]. Next, the produced DNA is purified by the means of filtration and chromatographic techniques. Tangential flow filtration (TFF), size-exclusion chromatography, hydrophobic interaction chromatography, and ion-exchange chromatography are the commonly used DNA purification methods. The next step in upstream processes is mRNA synthesis. In this step, the desired mRNA is synthesized from the template DNA by in vitro transcription using RNA polymerase. While different polymerases such as T7, T3, or SP6 can be used in this process, T7 is the most widely applied RNA polymerase in the mRNA industry due to its ability to synthesize long RNA transcripts [135,137]. Factors such as nucleotide triphosphates (NTPs) and magnesium (Mg) addition rate can highly affect the in vitro transcription reaction rate and the yield of mRNA; therefore, it must be controlled according to the consumption level in the reaction.

Downstream processes include mRNA purification which is a critical step in the production of therapeutic-grade mRNA. The raw in vitro synthesized mRNA contains a number of by-products and reaction reagents such as dsRNA, DNA template, NTPs, and enzymes. These impurities can lead to severe adverse effects and lower the translation efficiency of the produced mRNA. The removal of these impurities can be achieved on a large scale by the implementation of different techniques such as size exclusion chromatography (SEC) and ion pair reverse-phase chromatography (IPC) [135]. SEC is used to separate molecules according to their size; therefore, it can be used to separate mRNA molecules from larger and smaller impurities but not from the similar size impurities such as dsRNA which can be effectively removed by IPC. Other chromatographic techniques can be used in mRNA purification, including ion exchange chromatography (IEC), affinity chromatography, and cellulose-based chromatography, in addition to tangential flow filtration (TFF) which can be used to remove small size impurities [137]. These methods, despite being scalable and usable in the large-scale purification of mRNA, still have some drawbacks as each method can remove some specific impurities necessitating the use of other purification steps.

The next main step in manufacturing mRNA LNP-based vaccines is the association of mRNA with the LNP delivery system. The stability and efficacy of mRNA vaccines are greatly affected by the physicochemical properties of the LNPs such as their particle size, polydispersity, zeta potential, and mRNA loading efficiency. These properties are affected by the process and formulation parameters including the LNP preparation method, mRNA concentration, lipid concentration, lipids molar ratio, and lipid/mRNA ratio [10,138]. Therefore, the careful optimization of these factors is essential to ensure batch-to-batch consistency and product quality. In industry, LNPs are most commonly formulated using microfluidic techniques as they offer the precise control of the critical process parameters and product quality [139]. Staggered herringbone mixer (SHM) is a widely applied microfluidic mixer that can provide high-quality LNPs with a good reproducibility; however, it is not suitable for large-scale production due to its low output volume (in the scale of mL/h). Therefore, parallelization and modular systems are implemented to overcome this problem. Parallelization means using multiple mixing channels in the same microfluidic mixing chip to increase the yield of LNPs while maintaining their quality [140]. This technique has been reported to scale up LNP production from a small scale (mL/h) to a large industrial scale of L/h [9]. Despite the great efforts made in scaling up LNP production, the current techniques still face some challenges in meeting the required manufacturing scale needed for clinical and commercial purposes. Therefore, further research is required to provide alternative scale-up methods suitable for fulfilling the industrial requirements.

## 4. Preclinical and Clinical Studies in mRNA LNP-Based Cancer Vaccines

Recently, mRNA LNP-based cancer vaccines have become an attractive area of research, with many research groups working to maximize the efficacy of this technology in cancer treatment. Multiple preclinical and clinical studies have been published in the past 5 years, aiming at optimizing the delivery system and maximizing the anti-tumor immune response. In preclinical studies, researchers have investigated various LNP compositions, including different lipid components, new ionizable lipids, and specific organ targeting strategies, to enhance mRNA delivery and reduce off-target effects (Table 2). Some studies have focused on the incorporation of mRNA encoding a variety of immune-stimulating adjuvants, including TLR agonists (e.g., TLR2/6 agonists) [141], cytokine-based adjuvants (e.g., interleukin-12, interleukin-27) [142,143], and costimulatory molecules (e.g., CD40L) [144], to enhance the overall anti-tumor immune response. Moreover, strategies to target specific immune organs, such as draining lymph nodes (dLNs) or the spleen, have been explored to enhance the antigen presentation and immune activation [145,146,147,148]. Most of these studies have demonstrated the ability of mRNA LNP-based vaccines to induce strong immune responses, including the generation of tumor-specific T cells. In several preclinical models, mRNA LNP-based vaccines have shown efficacy in reducing tumor growth and even achieving complete tumor regression [141,143,147,148,149,150]. The vaccines have generally been well-tolerated in preclinical models, with acceptable survival rates.

On the other hand, despite the promising results achieved by preclinical research, only few clinical studies on mRNA LNP-based cancer vaccines are being conducted (Table 3). These clinical trials focused on the safety and efficacy of these vaccines in the treatment of different tumors, as a single therapy or in combination with other treatment approaches. Most of these clinical trials are still recruiting patients and have no published results [152,153].

## 5. Limitations, Challenges, and Future Directions in mRNA LNP-Based Cancer Vaccines

While mRNA LNP-based cancer vaccines have been very promising in these preclinical and early clinical studies, there are several limitations and challenges that must be addressed to unlock their real potential [158]. For instance, limited immune activation has been reported in preclinical and early clinical studies, limiting the translation of such a vaccine platform into clinical practices [69,159]. One of the main causes of the suboptimal immune response is the state of T cell exhaustion induced by the persistent antigen exposure in the tumor microenvironment. This exhaustion makes T cells less effective over time and limits the efficacy of the immune response [160]. Therefore, most of the current clinical and preclinical studies involve the co-administration of a checkpoint blockade inhibitor like anti-PD-1 therapy together with the vaccine candidate in order to reverse the state of T cell exhaustion and augment the vaccine action [161]. Additionally, other limitations can arise from the use of mRNA molecules themselves. For example, in vitro-transcribed mRNA molecules still have some defects in terms of their instability and degradation, innate immunogenicity, and translation efficiency [162]. mRNA is inherently unstable and susceptible to degradation by RNases, leading to a shorter half-life, decreased potency, and the need for a cold supply chain to preserve vaccine activity during storage and distribution [138]. Strategies to increase mRNA stability and translation efficiency, through the use of modified nucleosides and the proper optimization of the 5′ cap and poly(A) tail, still need to be further studied to gain a better understanding of the function and molecular effects of each modification [163,164,165]. Unmodified mRNA can also activate innate immune responses, thus reducing protein synthesis and possibly leading to potential side effects. Continued research is necessary to find the right modifications that will minimize the excessive immunogenicity while maintaining the adjuvant activity. By enhancing codon optimization and mRNA sequence design, it is possible to improve mRNA translation; however, there must still be further research to attain the maximum protein expression levels [166,167]. Incorporating self-amplifying or circular mRNA may further improve antigen expression levels and, thus, therapeutic vaccine efficacy [168,169,170].

Despite LNPs having been shown to be effective for the in vivo delivery of mRNA, their optimization for cancer immunotherapy is still a challenge. Issues, such as the targeting of immune cells, off-target accumulation in the liver, and improving antigen protein expression, need to be further optimized to induce a robust anti-tumor immune response [109,122,171]. The design of new ionizable lipids with improved transfection efficiency and the engineering of specific organ-targeting LNPs (SORT LNPs) for target delivery into lymphoid organs may help resolve these issues [132,146,172,173]. Also, the large-scale production of clinical-grade LNP-mRNA vaccines remains a challenge. Streamlining the manufacturing process and developing strict quality control measures are important for the widespread adoption of this technology [105]. In addition, LNPs themselves may also induce immune responses that limit repeated dosing or generate adverse events [174,175]. Ways for making LNPs less immunogenic, such as using stealth coatings or the engineering of less immunogenic lipid compositions, are currently under investigation [176].

The complex and immunosuppressive tumor microenvironment also poses a significant challenge to vaccine efficacy [177]. Tumor cells frequently display inhibitory molecules which have the ability to suppress immune responses, thus hindering the appropriate activation of anti-tumor T cells [178,179,180]. Another level of complexity comes from the fact that tumors are very heterogeneous regarding the antigen expression profile and immune landscape, which further complicates vaccine design and delivery [181,182]. These may incorporate, but will not be restricted to, the natural physical properties of tumors in the tumor microenvironment, which include a dense extracellular matrix, making it very difficult for immune cells to infiltrate and thus greatly limiting the effectiveness of anti-tumor vaccines [183,184]. Overcoming these challenges will require a thorough understanding of the TME and the development of new strategies to counteract the immune suppression and enhance the vaccine delivery. Moreover, tumor heterogenicity complicates the development of personalized anti-tumor vaccines, since optimal neoantigens will have to be identified differently in each patient [185]. Improvement in tumor sequencing methods, neoantigen prediction algorithms, and machine learning could make the selection of neoantigens more efficient [186,187]. In addition, the production of a tailored vaccine to each patient’s tumor is a time-consuming process. Increasing the efficiency of vaccine production processes and developing modular vaccine platforms could potentially support the acceleration of personalized vaccine production [188,189,190].

The future of mRNA LNP-based cancer vaccines holds great promise [191]. Applications of novel technologies, including machine learning and AI algorithms for mRNA design and sequence optimization, immunogenicity prediction, and neoantigen selection guidance, can accelerate the vaccine development process and enhance vaccines’ effectiveness [192,193,194,195,196]. In addition, novel ionizable lipids with improved safety profiles and enhanced endosomal escapes and targeted delivery capabilities can improve the efficacy and therapeutic outcomes of LNP-mRNA vaccines. Moreover, the combination of mRNA LNP-based vaccines with other kinds of immunotherapy, such as checkpoint inhibitors or adoptive cell transfer, may synergistically improve the antitumor immune response and clinical outcomes [197,198]. Ultimately, the comprehensive understanding of the interplay between the vaccine components, immune system, and tumor microenvironment will provide a background for the rational design of mRNA LNP-based cancer vaccines to achieve the full potential of such technology.

## 6. Conclusions

The concept of mRNA LNP-based cancer vaccines represents a huge potential for the transformation of cancer immunotherapy. The ability to induce an immune response against the encoded tumor antigens, together with the efficient intracellular delivery afforded by LNPs, makes it a quite promising technology at the forefront of cancer research. Although preclinical and early clinical data are encouraging, significant challenges are still to be addressed. Immense research is currently underway to improve vaccine efficacy and safety through advancements in mRNA design, including novel nucleoside modifications and codon optimization. These modifications can improve mRNA stability, translational efficiency, and reduce immunogenicity. As our understanding of mRNA structure and function deepens, the ability for the rational design of more effective vaccines increases. Overcoming obstacles such as tumor heterogeneity, immune evasion, and optimal vaccine delivery will require continued innovation. A comprehensive approach to developing improved mRNA design, LNP engineering, and the deep understanding of the tumor microenvironment is required to unlock the full potential of this technology. Now, as these challenges are being resolved, one can look forward to a time when the application of mRNA LNP-based cancer vaccines would be a cornerstone of personalized cancer therapy that would make a difference in patient outcomes.

## Figures and Tables

**Table 2:** Some of the reported preclinical studies on mRNA LNP-based cancer vaccines.

LNP Composition	mRNA	Tumor Model	Results	Ref.
MC3:DSPC:Cholesterol:PEG-DMA Molar ratio = 50:10:38.5:1.5	Unmodified, m1Ψ-modified, and self-amplifying mRNA, encoding hybrid HPV-16 E7 protein fused to HSV-1 gD	Mouse subcutaneous and mucosal tumor models of HPV-16–induced tumors.	− The 3 types of mRNA-LNPs induced protective immune responses after a single dose. − Induced stronger antigen-specific CD8+ T cell responses than recombinant protein and DNA vaccines	[151]
DALs:DOPE:Cholesterol:DMG-PEG Molar ratio = 20:30:40:0.75 DALs = new ionizable lipids containing di-amino groups with various head groups	Pseudouridine-5′-triphosphate-modified mRNAs encoding IL-12, IL-27, and GM-CSF	Subcutaneous mouse B16F10 melanoma model	− IL12 LNPs showed the highest tumor growth inhibition compared to IL27 LNPs and GM-CSF LNPs. − IL12 + IL27 LNPs had synergistic effects and completely inhibited tumor growth.	[142]
113-O12B:DOPC:Cholesterol:DMG-PEG Weight ratio = 16:4.8:3:2.4 113-O12B = synthetic ionizable lipid designed to direct LNPs distribution towards draining LNs.	mRNA encoding OVA protein	Mouse OVA-antigen-bearing B16F10 melanoma model	− 113-O12B LNPs showed increased expression in the LN compared with ALC-0315 LNPs − 113-O12B LNPs induced OVA-specific CD8+ T cells higher than ALC-0315 LNPs − 113-O12B LNPs completely inhibited tumor growth and prevented metastasis	[147]
Multi-armed ionizable lipid:DSPC:Cholesterol:PEG-lipid Molar ratio = 50:10:38:2	OVA (257-264)-luciferase-coding circular RNA	B16-OVA tumor lung metastasis, MC38-OVA subcutaneous model, and B16-OVA subcutaneous model	− LNPs induced robust antigen-specific T cell responses. − circRNA-LNPOVA-luc and m1Ψ mRNAOVA-luc-LNP significantly suppressed the tumor growth in the MC38-OVA model. − In the B16-OVA subcutaneous model, circRNA-LNPOVA-luc and m1Ψ mRNAOVA-luc-LNP completely suppressed the tumor growth. − circRNA-LNPOVA-luc and m1Ψ mRNAOVA-luc-LNP prevented lung metastasis after challenging mice with IV B16-OVA cells.	[149]
ALC-0315, DSPC, cholesterol and DMG-PEG Molar ratio = 50:10:38.5:1.5	OVA-encoding mRNA or CT26 neoantigen-encoding mRNA, co-encapsulated with Pam2Cys, a TLR 2/6 agonist.	E.G7-OVA subcutaneous C57BL/6 mouse model, and CT26 subcutaneous Balb/c mouse model.	− ALC-0315 pm LNPs induced IL-12 and IL-17 and other cytokines. − ALC-0315 pm LNPs eradicated the tumors in 50% of the mice with the E.G7-OVA tumor model and rejected tumor cells on rechallenge. − Prophylactically, inhibited tumor growth in 70% of the animals compared to 20% in the ALC-0315 LNPs without Pam2Cys. − In the CT26 tumor therapeutic model, ALC-0315 pm LNPs eradicated the tumors in 10% of the mice and delayed tumor growth.	[141]
TT3:DOPE:cholesterol:C14-PEG Molar ratio = 20:30:40:0.75	self-replicating RNA encoding IL-12-alb-lumican or IL-12-alb lumican is a collagen-binding protein, enhance retention in the tumors through binding to the tumor extracellular matrix	B16F10 melanoma model, YUMMER1.7 melanomas, and CT26 colon carcinomas, B16F10 tumor lung metastasis	− Induced tumor rejection and long-term survival in the majority of the animals. − LNP-Rep(IL-12-alb) induced high serum levels of IL-12, IFN-γ and other cytokines and chemokines. − Induced CD8+ T cells. − LNP-Rep(IL-12-alb) protected systemic immunity against lung metastasis	[143]
MC3:DSPC:Cholesterol:PEG-DMA Molar ratio = 50:10:38.5:1.5	OVA-encoding unmodified or m1Ψ-modified mRNA	Mouse B16F0-OVA subcutaneous model B16F0-OVA tumor lung metastasis model	− Unmodified mRNA) strongly induced IFN-I in BMDCs and BMDMs compared to m1Ψ-modified mRNA. − 100% m1Ψ-modified mRNA had efficient protein translation but did not induce the maturation of BMDCs, while unmodified mRNA induced DC maturation. − OVA-unmodified mRNA-LNP activated OVA-specific CD8+ T cells and stimulated CTL responses. − OVA-unmodified mRNA-LNP showed the highest tumor growth inhibition compared to m1Ψ-modified mRNA LNPs.	[150]

**Table 3:** Some current clinical trials of mRNA LNP-based cancer vaccines.

Sponsor	Cancer Type	Intervention	Target Antigen	Phase	NCT Member	Ref.
ModernaTX, Inc. (Cambridge, MA, USA)	Melanoma	mRNA-4157 and Pembrolizumab	Up to 34 neoantigens	1–2	NCT03897881	[154]
BioNTech SE (Mainz, Germany)	Colorectal cancer	RO7198457	mRNA encoding individual mutations	2	NCT04486378	[153]
BioNTech SE (Mainz, Germany)	HPV16+ head-and-neck squamous carcinoma	BNT113 and Pembrolizumab	HPV16-derived tumor antigens (E6 and E7 viral oncoproteins)	2	NCT04534205	[155]
Ludwig Institute for Cancer Research (NY, USA)	Non-small cell lung cancer	BI 1361849, Durvalumab, and Tremelimumab	NY-ESO-1, MAGE C1, MAGE C2, TPBG (5T4), survivin, MUC1	1–2	NCT03164772	[156]
BioNTech SE (Mainz, Germany)	Prostate cancer	BNT112 and cemiplimab	Fixed combination of five antigens commonly expressed in prostate cancer	1–2	NCT04382898	[157]
ModernaTX, Inc.	Solid tumors	mRNA-4157 and Pembrolizumab	Individualized cancer vaccine against up to 34 neoantigens	1	NCT03313778	[152]

## Data Availability

Not applicable.
